# MiR-206 is expressed in pancreatic islets and regulates glucokinase activity

**DOI:** 10.1152/ajpendo.00510.2015

**Published:** 2016-05-24

**Authors:** Manjula Vinod, Jay V. Patankar, Vinay Sachdev, Saša Frank, Wolfgang F. Graier, Dagmar Kratky, Gerhard M. Kostner

**Affiliations:** Institute of Molecular Biology and Biochemistry, Medical University of Graz, Graz, Austria

**Keywords:** miR-206, glucose sensing, insulin sensitivity, glucose tolerance, pancreatic islets

## Abstract

Glucose homeostasis is a complex indispensable process, and its dysregulation causes hyperglycemia and type 2 diabetes mellitus. Glucokinase (GK) takes a central role in these pathways and is thus rate limiting for glucose-stimulated insulin secretion (GSIS) from pancreatic islets. Several reports have described the transcriptional regulation of *Gck* mRNA, whereas its posttranscriptional mechanisms of regulation, especially those involving microRNAs (miR), are poorly understood. In this study, we investigated the role of miR-206 as a posttranscriptional regulator of *Gck*. In addition, we examined the effects of miR-206 on glucose tolerance, GSIS, and gene expression in control and germ line miR-206 knockout (KO) mice fed either with chow or high-fat diet (HFD). MiR-206 was found in *Gck-*expressing tissues and was differentially altered in response to HFD feeding. Pancreatic islets showed the most profound induction in the expression of miR-206 in response to HFD. Chow- and HFD-fed miR-206KO mice have improved glucose tolerance and GSIS but unaltered insulin sensitivity. In silico analysis of *Gck* mRNA revealed a conserved 8-mer miR-206 binding site. Hence, the predicted regulation of *Gck* by miR-206 was confirmed in reporter and GK activity assays. Concomitant with increased GK activity, miR-206KO mice had elevated liver glycogen content and plasma lactate concentrations. Our findings revealed a novel mechanism of posttranscriptional regulation of *Gck* by miR-206 and underline the crucial role of pancreatic islet miR-206 in the regulation of whole body glucose homeostasis in a murine model that mimics the metabolic syndrome.

A surfeit of nutrients combined with a sedentary lifestyle has resulted in an alarming increase in the rate of obesity-associated diseases such as type 2 diabetes (T2DM) and metabolic syndromes (30). T2DM is characterized by hyperglycemia owing to inadequate amounts of glucose-stimulated insulin secretion (GSIS) combined with profound peripheral insulin resistance. T2DM is also a major trigger for cardiovascular diseases (18). Genome-wide association and candidate gene studies have identified more than 50 loci associated with T2DMand obesity (19) that underline the complex nature of glucose homeostasis. However, such approaches are largely exclusive to the coding regions of the genome, and regulatory influences of the noncoding genomic regions on disease outcomes are neglected.

Maintenance of glucose homeostasis is mediated by a concerted action involving multiple regulatory mechanisms. More recently, the role of noncoding RNAs, especially micro-RNAs (miRs), in regulating several steps of glucose metabolism has been discovered (24). Various miRs are known to influence multiple aspects of whole body glucose homeostasis and insulin sensitivity. GSIS, one of the most crucial steps in glucose utilization, is also subject to such fine-tuning via β-cell-specific miRs (7). Impaired GSIS is essentially the key to β-cell failure and T2DM, with aberrant miR profiles being implicated as an important etiological factor.

The function of pancreatic β-cells is facilitated by the rate-limiting glucose sensor glucokinase (GK) and the low-affinity glucose transporter GLUT2. GK is expressed in liver and various neuroendocrine cells such as pancreatic islets, small intestine, and brain (14, 23). The most intriguing evidence supporting the GK-glucose sensor concept arises from the functional consequences of *Gck* mutations in humans. Most of these mutations manifest as autosomal dominant inherited syndromes, including *Gck*-linked permanent neonatal diabetes, mature onset diabetes of the young, and others (8). Loss of *Gck* in rodents leads to severe hyperglycemia and further emphasizes the role of GK in the regulation of glucose homeostasis (17).

The expression levels of GK in the liver and pancreatic islets are stringently controlled by tissue-specific promoters and pathways triggered by insulin and glucagon (3). Binding sites for PPARγ, RXRα, SREBP1c, PDX-1, and HNFs have been identified in the corresponding promoter. More recently, additional transcriptional factors such as LRH-1 and BETA2/NeuroD1 have been reported to induce the expression of GK in a tissue-specific manner (15, 20). GK is also posttranscriptionally regulated by a high-affinity GK regulatory protein (GKRP), which undergoes fructose phosphate and glucose-mediated shuttling between the nucleus and the cytoplasm (9, 10). In addition, GK activity is posttranscriptionally controlled by various mechanisms (6, 21).

Several miRs have predicted binding sites in the 3′-untranslated region (3′-UTR) of *Gck*. However, direct evidence of a miR that targets *Gck* mRNA is lacking. Here, we provide evidence that miR-206 downregulates the expression of *Gck* by specifically targeting the 3′-UTR of *Gck* mRNA. miR-206 is expressed in multiple tissues in mice, including pancreatic islets. The transcript levels of miR-206 are upregulated in islets and liver of high-fat diet (HFD)-induced diabetic wild-type mice compared with chow-fed controls. A resistance to impaired glucose metabolism in response to a HFD was observed in miR206-knockout mice. The improvements included improved glucose and insulin tolerance, increased insulin secretion, higher *Gck* mRNA expression in islets, and increased GK activity in pancreas and liver.

## Materials and Methods

### Animals and diet

All animal experiments were carried out in accordance with the guidelines set by the Division of Genetic Engineering and Animal Experiments and were approved by the Austrian Federal Ministry of Science, Research, and Economy (Vienna, Austria). Mice used in this study were all on a 29SvEv-C57BL/6 mixed background. MiR-206-knockout (miR-206KO) mice were originally obtained from Dr. Eric N. Olson’s laboratory (Department of Molecular Biology, University of Texas Southwestern Medical Center). All animals were fed ad libitum with normal chow (NC) diet (11.9% calories from fat; Ssniff, Soest, Germany) and maintained in a 12:12-h light-dark cycle in a temperature-controlled environment. To study diet-induced changes, individually housed male miR-206KO mice and control littermates (aged 5 wk) were fed a high-fat/high cholesterol diet (HFD) (D12492 sniff; 60% calories from fat, 21% calories from carbohydrates, and 19% calories from protein; in addition, we added 1 g of cholesterol to 99 g of dry chow). Food intake was measured every 2 wk during the feeding period and was calculated as grams per day per mouse.

### Murine islet isolation

Murine islets were isolated from age- and sex-matched control and miR-206KO mice, as described (31). Briefly, mice were euthanized by cervical dislocation. The common bile duct was cannulated using a 27G needle, and 2 ml of ice-cold Liberase II (Roche Diagnostics, Basel, Switzerland) was injected immediately. The perfused pancreas was dislodged from the intestine, spleen, and stomach. To facilitate complete digestion, the pancreas was further incubated in a water bath preheated at 37°C for predetermined time intervals (batch variation, average time 12 min and 30 s). After digestion, islets were purified by filtration and gradient separation. Islets were maintained in RPMI (GIBCO; Life Technologies, Carlsbad, CA) supplemented with 10% fetal bovine serum and 100 U/ml penicillin-streptomycin (GIBCO) for 24 h, and healthy islets were hand-picked for experiments.

### Quantitative real-time PCR analysis

Quantitative real-time PCR was carried out as described earlier (28). Data were analyzed using the open access software Relative Expression Software Tool - REST 2010 (http://www.gene-quantification.com/download.html) (22).

### Glucokinase activity assay

GK activity was measured in liver and islets obtained from control and miR-206KO mice. One hundred milligrams of liver tissue was homogenized in RIPA buffer and centrifuged at 13,000 *g* at 4°C. The supernatant was used for measuring GK activity. Islet GK activity was determined from ~100 hand-picked islets isolated from control and miR-206KO mice. The islets were washed in PBS, lysed in RIPA buffer, sonicated, and centrifuged. The supernatant was used to analyze GK activity as described (4).

### In vivo GSIS

To quantify the amount of insulin secreted into the plasma in response to glucose, a solution of d-glucose(2 g/kg body wt) was injected intraperitoneally into control and miR-206KO mice. Blood was collected from the retro-bulbar plexus 0, 15, 30, and 60 min after the injection. Plasma was isolated and analyzed for the amount of secreted insulin.

### Microarray meta-analysis

We meta-analyzed gene expression profiles of diabetic vs. nondiabetic human islets using a publicly available data set (accession no. GDS3882) (5). Meta-analyses were carried out using the freely available software package BRB-Array Tools version 4.3.2 that was developed under the direction of R. Simon at the Biometric Research Branch, National Cancer Institute (http://linus. nci.nih.gov/BRB-ArrayTools.html). A list of predicted miR-206 target genes was generated from data obtained from Targetscan (http://www.targetscan.org/). The microarray data sets were normalized to exclude threshold intensities of <10, with the median array as reference. All genes with >50% missing replicate values on each array were excluded from all analyses. All data obtained were sorted and compared using Microsoft Excel. Scatterplots were generated using BRB Array Tools, and the highlighting gene set function within Array Tools was used to discriminate miR-206 target genes.

### Intraperitoneal glucose tolerance test

Intraperitoneal glucose tolerance test (IPGTT) was carried out as described (11). Prior to IPGTT being performed, the animals were starved overnight (12 h), and a solution of d-glucose (2 g/kg body wt) was injected intraperitoneally into control and miR-206KO mice. Blood was drawn from the tail vein for glucose measurements at the designated time points.

### Insulin tolerance test

To compare the insulin sensitivity in randomly fed control and miR-206KO mice an insulin tolerance test (ITT) was performed as described earlier (11).

### Quantification of pancreatic insulin content

Pancreatic insulin content was measured from 12-wk-old male NC or HFD-fed control and miR-206KO mice after overnight fasting. Posteuthanization, pancreata were isolated, weighed, and homogenized in acid-ethanol solution (1.5% HCl in 70% ethanol) overnight at −20°C, followed by neutralization with 1 M Tris, pH 7.5. Insulin levels were quantified by ELISA (Mercodia, Uppsala, Sweden) according to the manufacturer’s protocol. Insulin levels were normalized to pancreatic weight. Insulin concentrations in plasma samples after GSIS and randomly fed or fasted control and miR-206KO mice were determined using the same ELISA.

### Luciferase reporter assays

The wild-type *Gck*-3′-UTR with intact miR-206 seed sequence and the *Gck*-3′-UTR with scrambled miR-206 seed sequence were cloned downstream of a luciferase reporter gene into the pMIR-REPORT-miR expression reporter construct (Ambion, Carlsbad, CA) and β-gal expression vector and cotransfected with the miR-206-expressing vector into COS-7 cells using Fugene HD (Roche, Basel, Switzerland) according to the manufacturer’s protocol. The relative luciferase activity was measured using the luciferase assay system (Promega, Madison, WI). β-Gal activity was measured using the β-Galactosidase Enzyme Assay System (Promega). The luciferase activity in each sample was normalized to β-gal activity to obtain relative luciferase units (RLU). %RLU in samples transfected with the wild-type *Gck*-3′-UTR was calculated to the scrambled *Gck*-3′UTR.

### Biochemical assays

Liver glycogen measurements were performed according to Adamo and Graham (1). All protein concentrations were measured using a standard BCA technique. Plasma lactate from HFD-fed control and miR-206KO mice was determined using lactate colorimetric assay kit (BioVision, San Francisco, CA) according to the manufacturer’s instructions.

### Statistics

For IPGTT, ITT, and GSIS experiments, differences between the control and miR-206KO groups were compared using the two-parameter analysis of variance (2-way ANOVA). Differences in glycogen and lactate content and GK activity were calculated using a two-tailed, unpaired Student’s *t*-test. All statistical analyses were performed using GraphPad Prism 5.0. Relative fold change in transcript levels of the indicated genes was quantified by randomized pairwise allocation analysis using the open access software Relative Expression Software Tool - REST 2010 (http://www.gene-quantification.com/download.html)(22).

## Results

**Fig. 1:**
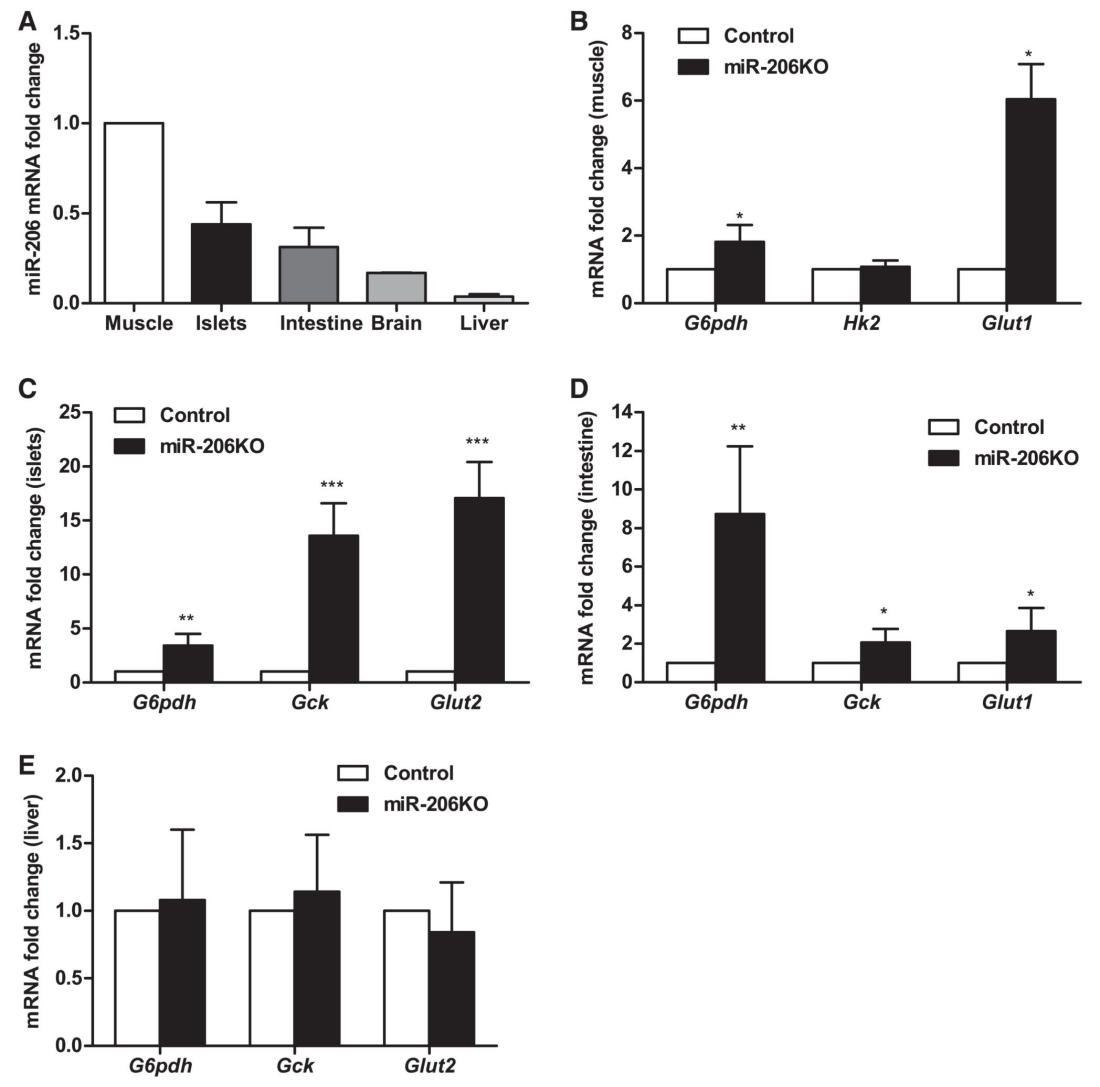
miR-206 expression in murine tissues. *A*: abundance of miR-206 in muscle, islets, intestine, brain, and liver relative to U6 sno-RNA measured by real-time PCR (*n* = 5/group). *B*–*E*: mRNA abundance of key glucose homeostasis genes in muscle (*B*), pancreatic islets (*C*), intestine (*D*), and liver (*E*), relative to hypoxanthine-guanine phosphoribosyl transferase (*Hprt*) expression, measured by real-time PCR (*n* = 5). Data represent means ± SE. *P < 0.05, **P < 0.01, and ***P < 0.001 by randomization tests with a pairwise reallocation. *Hk2*, hexokinase; *Gck*, glucokinase gene; *Glut1* and -*2*, glucose transporter 1 and 2, respectively.

*miR-206 is expressed in multiple murine tissues and impacts levels of glycolytic transcripts*. Conventionally, miR-206 is considered a muscle-specific miR. We also found the highest expression of miR-206 in muscle, yet the expression was not restricted to it. Relative to muscle, miR-206 expression was 44% in islets, 31% in intestine, and 17% in brain, but only 3.7% in the liver (Fig. 1*A*). A recent report on the regulation of glycolytic genes by miR-206 prompted us to investigate the relative transcript levels of genes involved in glucose homeostasis in the muscle, islets, intestine, and liver of miR-206KO (25). In miR-206-knockout muscles, transcripts of important glycolytic genes such as glucose-6-phosphate dehydrogenase (*G6pdh*) and glucose transporter 1 (*Glut1*) were increased by 1.8- and 6.0-fold, respectively (Fig. 1*B*), whereas hexokinase 2 (*Hk2*) mRNA expression was comparable in miR-206KO and control mice.

In miR-206KO islets, mRNA expression of *G6pdh, Gck*, and *Glut2* was elevated by 3.4-, 13.6-, and 17.0-fold, respectively (Fig. 1*C*). In the intestine, the transcript levels of *G6pdh, Gck*, and *Glut1* were increased in miR-206KO mice by 8.7-, 2.0-, and 2.6-fold, respectively, compared with controls (Fig. 1*D*). On the chow diet, hepatic transcript levels of *G6pdh, Gck*, and *Glut2* were comparable in miR-206KO and control livers (Fig. 1*E*), which was probably due to low hepatic miR-206 expression. These data indicate that miR-206 may be involved in regulating glycolytic gene expression in tissues with significant miR-206 abundance.

### Genetic deletion of miR-206 improves glucose tolerance and GSIS

**Fig. 2:**
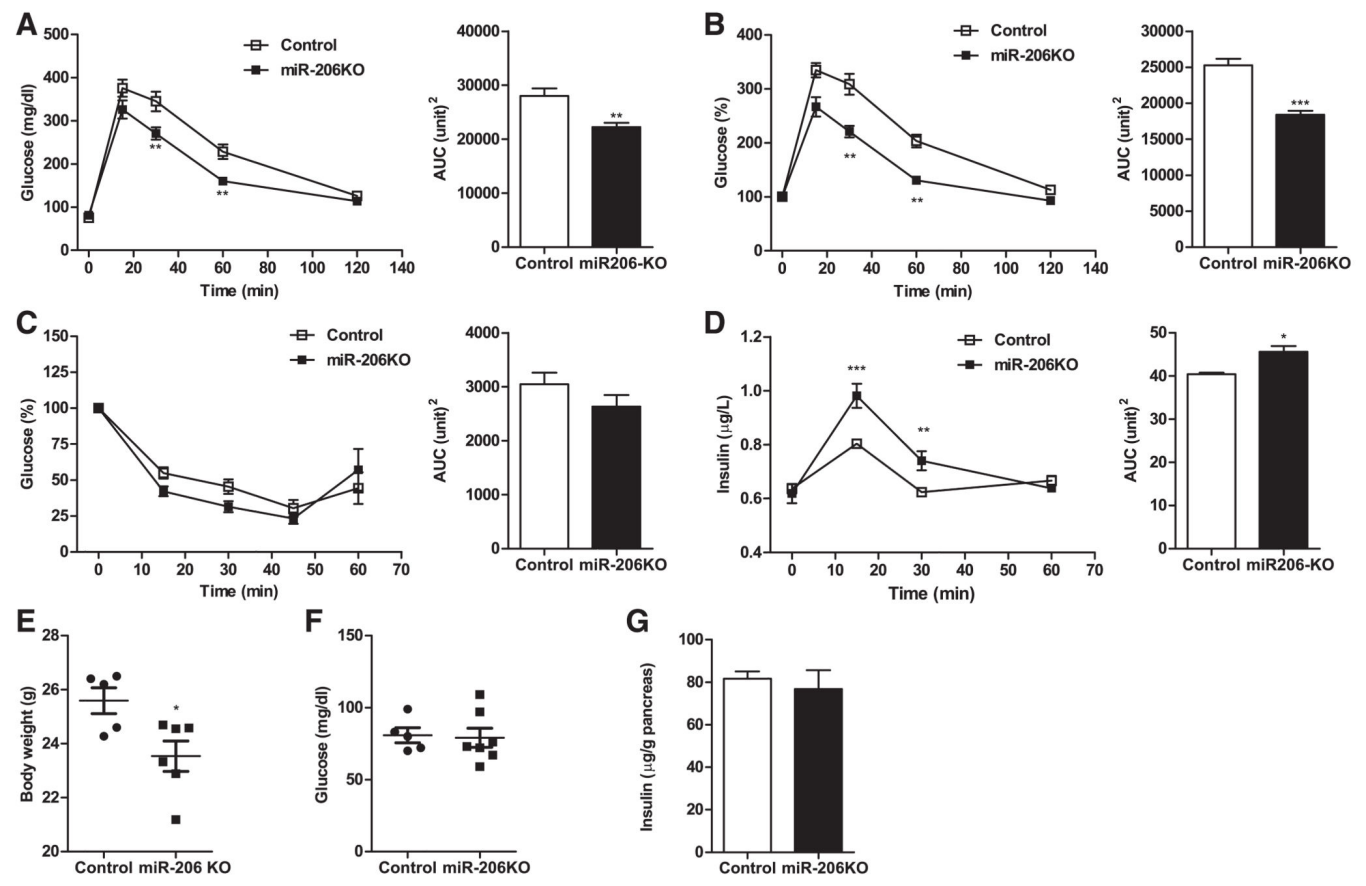
Glucose (GTT) and insulin tolerance tests (ITT) and insulin release in chow diet-fed mice. *A* and *B*: glucose excursions in response to 2 g/kg ip injected glucose and corresponding area under the curve (AUC) in mg/dl and in *%time 0* (%T0), respectively (*n* = 6/group). *C*: blood glucose decrease in response to 0.75 U ip injected insulin and corresponding AUC (*n* = 6/group). *D*: insulin secretion in response to 2 g/kg ip injected glucose and corresponding AUC (*n* = 3–4) in control (open bars and □) and MiR-206-knockout (miR-206KO) mice (black bars and ■) fed chow diet. *E*–*G*: body weights (*E*), fasting glucose concentrations (*F*), and pancreatic insulin content (*G*) in 12-wk-old male control and miR-206KO mice. Data represent means ± SE. *P < 0.05, **P < 0.01, and ***P < 0.001 by 2-way ANOVA for GTT and ITT and Student’s unpaired *t*-test for AUC, body weight, and fasting glucose measurements.

To investigate the physiological impact of the changes in glycolytic gene expression, we characterized in vivo glucose handling in miR-206KO and control mice. MiR-206KO mice showed significantly lower plasma glucose excursions 30 and 60 min post-glucose injection and a significant reduction in AUC, indicating increased glucose tolerance (Fig. 2, *A* and *B*). However, the peripheral insulin tolerance was unaffected by miR-206 deficiency (Fig. 2*C*).

We hypothesized that due to induction in islet *Gck* mRNA, miR-206KO mice may have an increased insulin secretion. Measurement of GSIS revealed that miR-206KO mice secreted more insulin 15 and 30 min post glucose injection by 22% and 20% compared with controls. This corresponded to an increase of 13% in area under the curve (AUC; Fig. 2*D*). miR-206KO mice had reduced body weight (Fig. 2*E*), whereas fasting glucose levels were comparable between control and miR-206KO mice (Fig. 2*F*). The observed increase in insulin secretion was independent of the total insulin content in miR-206KO pancreas (Fig. 2*G*). These findings indicate that miR-206 does not affect the regulatory mechanism of insulin production but modulates insulin secretion.

### In human type 2 diabetic islets the majority of dysregulated miR-206 predicted targets are repressed

**Fig. 3:**
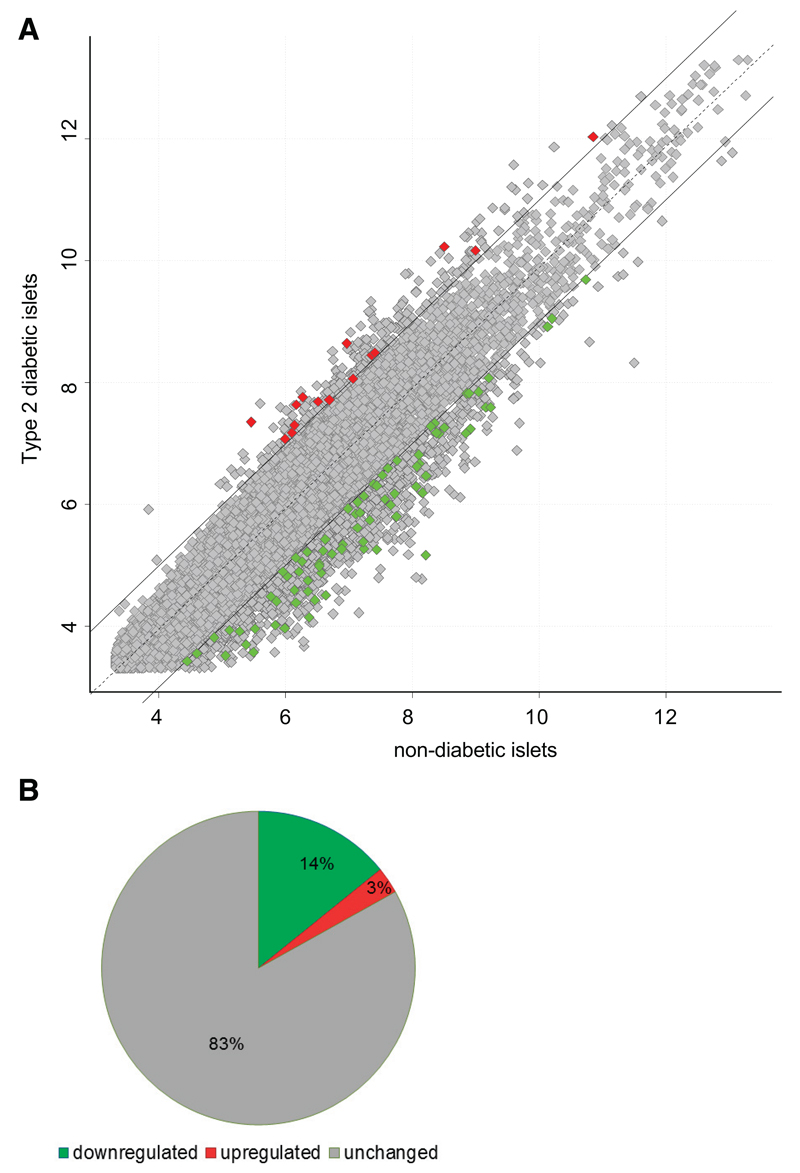
Meta-analysis of microarray data for miR-206 targets in type 2 diabetes mellitus human islets. *A*: scatter plot showing the meta-analysis of publically available microarray data from diabetic and nondiabetic islets. Regulated miR-206 targets in diabetic islets (red squares, upregulated; green squares, down-regulated). *B*: pie chart of predicted miR-206 genes represented in the meta-analysis showing %regulated (red), down-regulated (green), and nonregulated (gray) genes.

We meta-analyzed publically available microarray data to identify differences in gene expression profiles of predicted miR-206 targets in T2DM vs. nondiabetic islets. Analyses revealed that only a small proportion of the predicted miR-206 targets (17%) were differently regulated in T2DM compared with nondiabetic islets. However, a staggering 90% of these 17% genes were down-regulated in T2DM islets (Tables 1 and 2). This finding indirectly implies that miR-206 may be upregulated in the islets of diabetic individuals (Fig. 3, *A* and *B*).

### miR-206KO mice are protected from high-fat diet-induced impairment in systemic glucose homeostasis

**Fig. 4:**
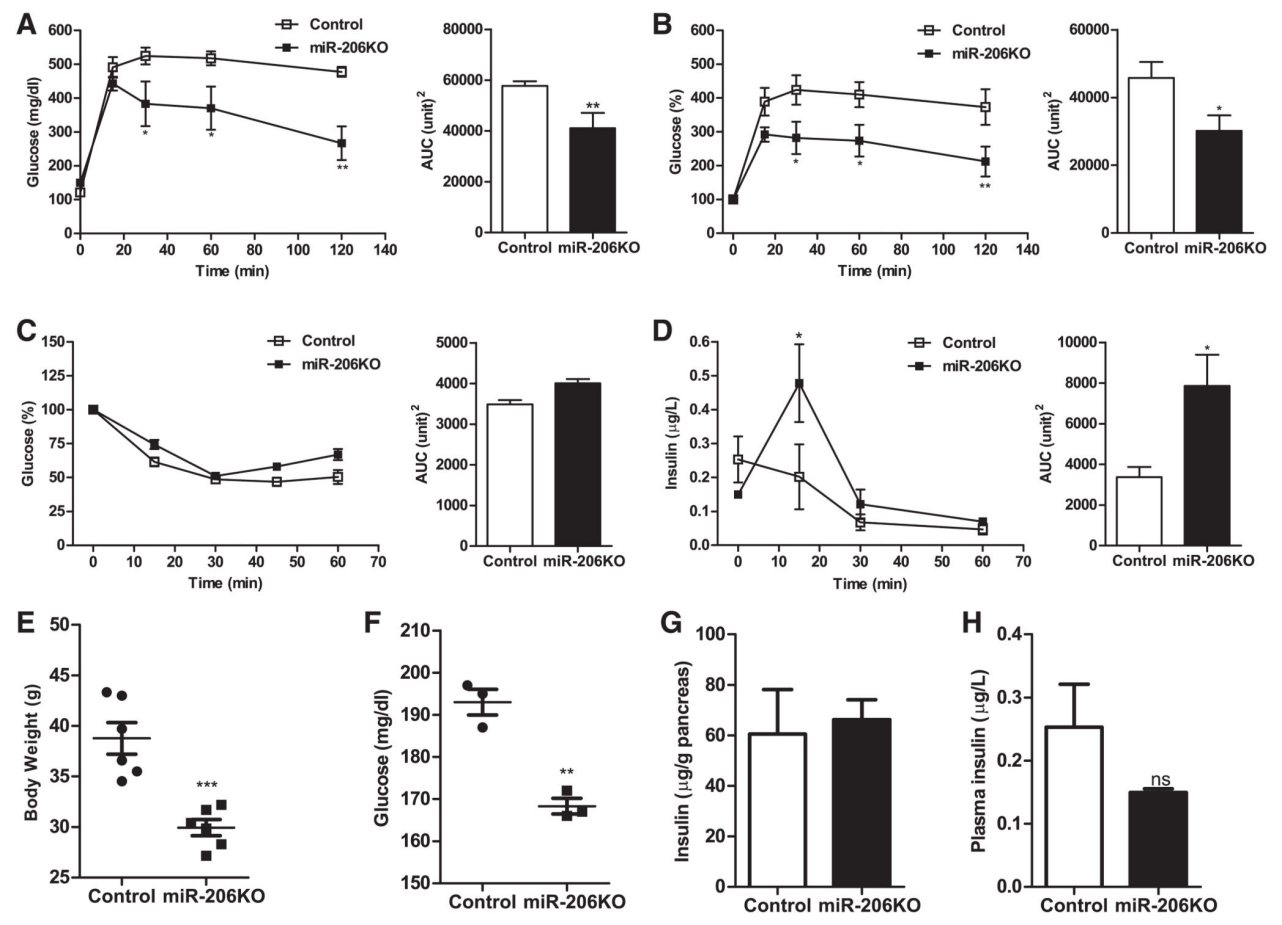
Glucose (GTT) and insulin tolerance tests (ITT) and insulin release in high-fat diet (HFD)-fed mice. *A* and *B*: glucose excursions in response to 2 g/kg ip injected glucose and corresponding AUC in mg/dl and %T0, respectively (*n* = 6/group). *C*: blood glucose concentrations in response to 0.75 U ip injected insulin and corresponding AUC (*n* = 4/group). *D*: insulin secretion in response to 2 g/kg ip injected glucose and corresponding AUC (*n* = 4/group) in control (open bars and □) and miR-206KO mice (black bars and ■) fed HFD for 20 wk. *E*: body weights of HFD-fed control and miR-206KO mice after 14 wk of feeding (*n* = 6/group). *F*: fasting glucose levels in HFD-fed control and miR-206KO mice after 20 wk of feeding (*n* = 3). *G*: pancreatic insulin content after 20 wk of HFD feeding (*n* = 4/group). *H*: circulating plasma insulin levels in randomly fed control and miR-206KO mice (*n* = 4/group). Data represent means ± SE. *P < 0.05, **P < 0.01, and ***P < 0.001 by 2-way ANOVA for GTT and ITT and Student’s unpaired *t*-test for AUC, body weight, insulin, and fasting glucose measurements.

In view of the results from meta-analysis and the improved GSIS in miR-206KO mice, we next investigated the role of miR-206 in a murine model of metabolic syndrome. Since it is known that high-fat diet (HFD) alters the expression of glycolytic genes and induces a metabolic syndrome in mice, we challenged miR-206KO and control mice with such a diet. In fact, glucose tolerance was significantly greater in HFD-fed miR-206KO compared with control mice 15, 30, and 60 min post-glucose injection, with a 34% lower AUC (Fig. 4, *A* and *B*).

Independent of the genotype, the maximum reduction in plasma glucose during ITT was 55% in HFD-fed mice (Fig. 4C), whereas chow diet-fed animals achieved 77% (Fig. 2*C*). This result indicates overall insulin resistance in response to HFD. However, the insulin tolerance was comparable between HFD-fed miR-206KO and control mice.

The 20-wk HFD-feeding paradigm lowered insulin levels in both genotypes. We failed to induce a significant insulin response to ip injected glucose in HFD-fed control mice. Two-way ANOVA did not yield significance in insulin released by the two genotypes. However, relative to baseline, miR-206KO mice showed a significant threefold induction (unpaired *t*-test, *P* = 0.048) in insulin secretion 15 min post-ip glucose injection compared with relative secretion in controls at the same time point (Fig. 4*D*). Notably, the overall AUC for the GSIS in miR-206KO was increased significantly by 2.3-fold (Fig. 4*D*). Body weight (Fig. 4*E*) and fasting glucose concentrations (Fig. 4*F*) were markedly reduced in miR-206KO compared with control mice after HFD feeding. However, compared with chow diet-fed mice, pancreatic insulin content was similar in both genotypes (Fig. 4*G*). Circulating plasma insulin levels in randomly fed control and miR-206KO mice were not significantly different (Fig. 4*H*). Thus, increased GSIS in miR-206KO mice is independent of insulin content in the pancreas, indicating that another mechanism is operative in miR-206KO mice.

### High-fat diet differentially regulates the expression of mir-206 and glycolytic genes in different tissues

**Fig.5:**
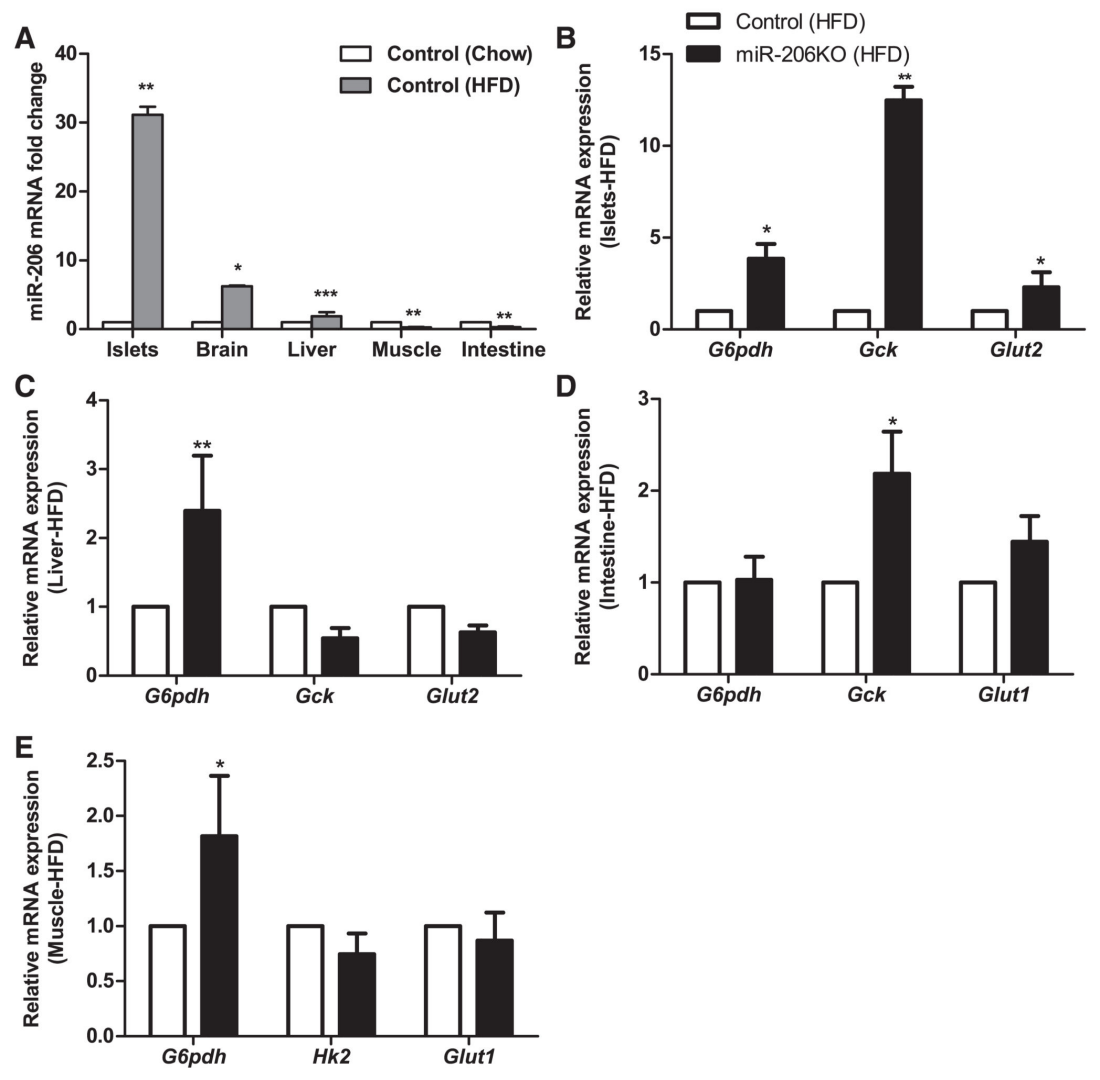
Expression of miR-206 and glycolytic genes in HFD-fed mice. *A*: relative abundance of miR-206 in islets, brain, liver, muscle, and intestine relative to U6 sno-RNA measured by real-time PCR (*n* = 5/group). *B*–*E*: mRNA abundance of *G6pdh, Gck*, and *Glut2* in islets (*B*) and liver (*C*), *G6pdh, Gck*, and *Glut1* in intestine (*D*), and *G6pdh, Hk2*, and *Glut*1 (E) in skeletal muscle of control (open bars) and miR-206KO mice (black bars) fed HFD for 20 wk (*n* = 5/group) relative to *Hprt* expression measured by real-time PCR. Data are presented as means ± SE. *P < 0.05, **P < 0.01, and ***P < 0.001 by randomization tests with a pairwise reallocation.

Next, we elucidated the impact of miR-206 deletion on changes in gene expression stimulated by HFD in multiple tissues. Interestingly, miR-206 expression itself was subject to regulation by HFD and was upregulated by 31-, 6.2-, and 1.8-fold in islets, brain, and liver, respectively, compared with the expression in chow diet-fed controls. In contrast, HFD feeding resulted in repression of miR-206 transcripts by 76 and 69% in skeletal muscle and intestine, respectively (Fig. 5*A*).

Islets showed the most profound induction in the expression of miR-206 in response to HFD (Fig. 5*A*). Consistent with what we observed in chow diet-fed mice, transcript levels of *G6pdh, Gck*, and *Glut2* were elevated by 3.8-, 12.5-, and 2.3-fold, respectively (Fig. 5*B*). Due to the low expression of miR-206 in the liver, we expected only marginal effects of miR-206 in regulating hepatic glycolytic genes. mRNA expression of hepatic *Gck* and *Glut2* were unaltered, whereas *G6pdh* transcript levels were 2.4-fold higher in miR-206KO compared with control mice (Fig. 5*C*). Interestingly, unlike in islets, intestinal miR-206 expression was repressed upon HFD. Therefore, we tested whether expression of glycolytic genes varies inversely with respect to miR-206 levels. Transcript levels of intestinal *Gck* were upregulated 2.1-fold in HFD-fed miR-206KO mice compared with controls (Fig. 5*D*), whereas *G6pdh* and *Glut1* mRNA expression were unchanged (Fig. 5*D*). In muscle, HFD-feeding resulted in a 1.8-fold increase in *G6pdh* mRNA expression, whereas hexokinase 2 (*Hk2*) and *Glut1* levels were comparable between both genotypes (Fig. 5*E*).

### Gck is a direct target of miR-206, and genetic deletion of miR-206 affects GK activity in pancreatic islets

**Fig. 6:**
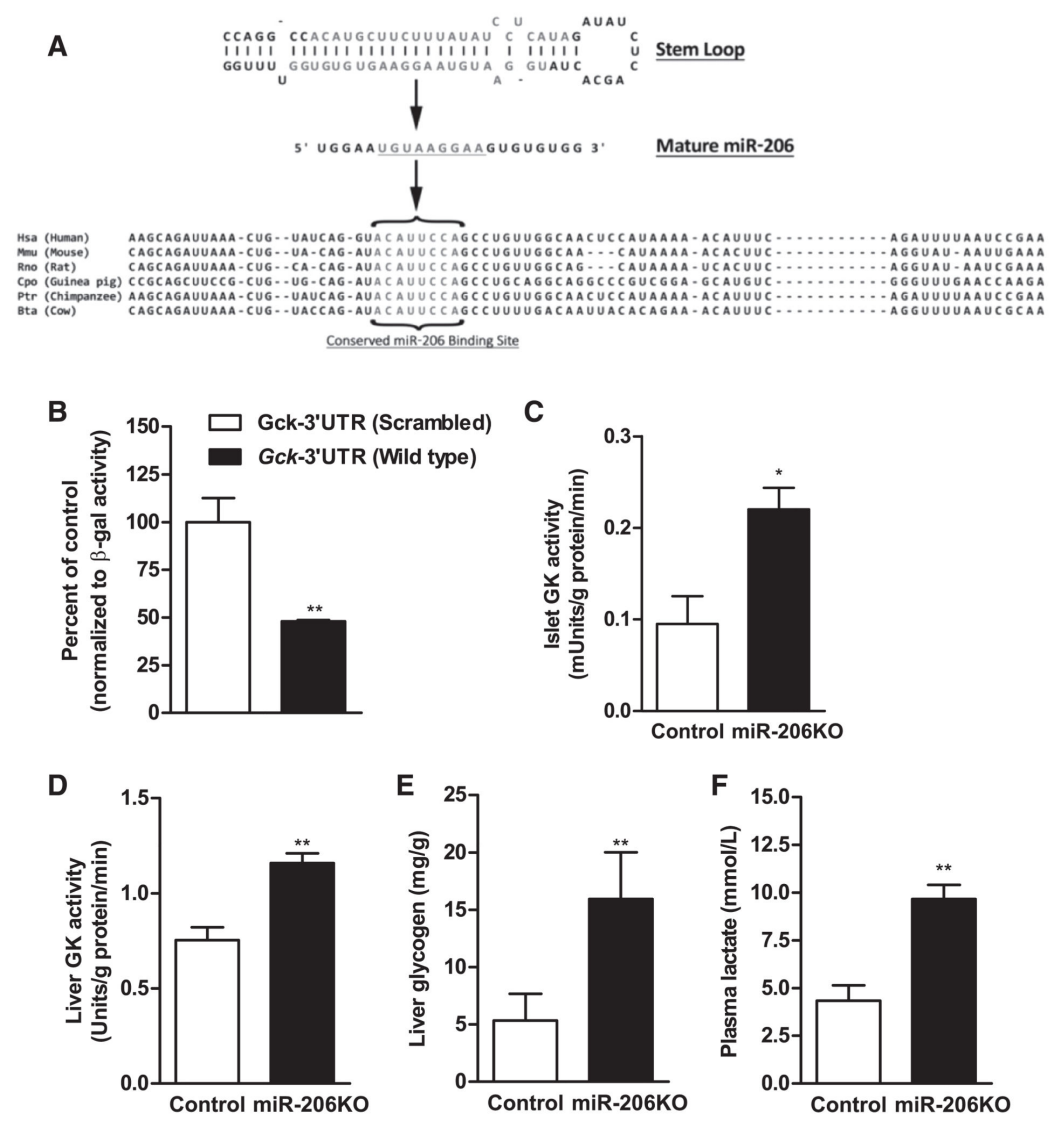
miR-206 targets *Gck* mRNA. A: stem-loop structure of pre-miR-206 with the seed sequence of miR-206 (underlined letters) and the conserved miR-206 binding site in *Gck* 3′-untranslated region (UTR) from the indicated species (gray letters). *B*: relative luciferase activity of cells expressing recombinant luciferase mRNA harboring the *Gck* 3′-UTR with either wild-type miR-206 seed sequence (black bar) or a scrambled miR-206 seed sequence (open bar) as control. *C* and *D*: glucokinase (GK) activity measured in isolated islets (*C*) and liver (*D*) from control (open bar) and miR-206KO mice (black bar) (*n* = 4/group) fed chow diet. *E* and *F:* liver glycogen content (*E*) and plasma lactate concentrations (*F*) in control (open bar) and miR-206KO (black bar) mice fed HFD for 2 wk. Data are presented as means ± SE. *P< 0.05 and **P < 0.01, Student’s *t*-test.

Our results have shown so far that *Gck* mRNA expression varied most consistently, in accordance with miR-206 transcript levels. Thus, we searched for posttranscriptional mechanisms of miR-206-mediated targeting of *Gck* by in silico analysis. TargetScan analysis of *Gck* mRNA revealed a conserved 8-mer target site for miR-206 with a favorable binding energy of (−) 31.0 kcal/mol (Fig. 6*A*). We next performed luciferase reporter assays in COS-7 cells cotransfected with a miR-206 expression plasmid and the *Gck*-3′-UTR clone containing the wild-type miR-206 seed sequence or a control *Gck*-3′-UTR clone containing a scrambled miR-206 seed sequence. The results revealed a 52% decrease in luciferase activity in the *Gck*-3′UTR with intact miR-206 seed sequence compared with the control clone with scrambled miR-206 seed sequence (Fig. 6*B*).

To functionally validate this observation, we measured GK enzyme activity in pancreatic islets and liver homogenates. GK activity was increased 2.3-fold in miR-206KO compared with control islets (Fig. 6*C*). GK activity was also 1.5-fold higher in miR-206KO liver compared with controls (Fig. 6*D*). An increase in hepatic GK activity directly translates into increased liver glycogen. As predicted, HFD-fed miR-206KO livers showed a threefold increase in glycogen content compared with HFD-fed controls (Fig. 6*E*). Plasma lactate levels are a covariant with systemic GK activity (26) and were 2.2-fold higher in miR-206KO mice (Fig. 6*F*).

## Discussion

The principle findings of our study are as follows: *1*) miR-206 is expressed not only in muscle but also in islets, brain, intestine, and liver, and its expression is tissue specifically altered in response to HFD; 2) miR-206 targets *Gck* and represses its activity; *3*) loss of miR-206 affects important glycolytic genes involved in maintaining systemic glucose homeostasis; and *4*) miR-206KO mice have improved glucose tolerance due to an increase in GK activity and insulin secretory capacity in response to glucose.

miRs have emerged as key posttranscriptional regulators of the majority of human genes; they respond to physiological and pathological stress and function mainly to maintain the systemic homeostatic balance (2). Pancreatic β-cells and insulin-sensitive tissues express a distinct set of miRs, and an increasing body of evidence illustrates the role of miRs in glucose homeostasis (7). Several miRs such as miR-7a, miR-29a/b/c, miR-30d, and miR-338p are implicated in pathological processes such as inflammation-mediated β-cell apoptosis, impaired insulin secretion, and insulin production (12, 24, 29).

Very recently, Singh et. al. (25) reported the reprogramming of glucose metabolism in cancer cells mediated by sustained activation of nuclear factor erythroid-2-related factor 2 (NRF2). NRF2 represses miR-206 expression, resulting in increased expression of its target genes involved in the pentose phosphate pathway, tricarboxylic acid cycle, and fatty acid synthesis. Since cancer cells predominantly express hexoki-nase, the regulation of *Gck* in this context was not studied. Recent studies have also shown that dietary metabolites influence the expression of β-cell miRs, which in turn orchestrate the changes in gene expression of several targets that influence β -cell function.

Here, we demonstrate that HFD leads to differential regulation of miR-206 in a tissue-specific manner. We describe a previously unknown role of miR-206 in the regulation of glucose homeostasis by modifying expression and activity of the key glycolytic enzyme GK. Glucose sensing in the pancreas is vital for maintaining physiological plasma concentrations of glucose, and β-cell GK has been recognized as a glucose sensor of paramount importance. In hepatocytes, GK serves as a mediator of glucose clearance and catalyzes and regulates important steps in glycogen biosynthesis.

Liver GK activity serves as a cardinal metabolic switch between fed and fasted states of carbohydrate metabolism. During the fed state, increased insulin-mediated uptake and phosphorylation of circulating glucose into liver by GK facilitate the storage of glucose as glycogen. Although the expression of hepatic *Gck* was only marginally influenced by miR-206, GK activity was increased in the liver of miR-206KO mice, leading to higher hepatic glycogen content. This increase in liver glycogen of HFD-fed miR-206KO mice may also be attributed to increased *G6pdh* mRNA expression in miR-206KO liver.

Murine *Gck* mRNA has two transcript variant isoforms, 1 and 2, with different 5′-UTR regions and identical 3′-UTR regions. *Gck* transcript variant 1 is expressed in pancreatic β-cells, whereas transcript variant 2 is expressed in the liver. In silico predictions revealed that miR-206 could putatively bind to both transcript variants and regulate *GK* repression via its target site in the *Gck* 3′-UTR. We validated the functionality of the predicted miR-206 target site in the 3′-UTR region of the *Gck* by miR-206-reporter luciferase assay. The luciferase activity in cells transfected with the *Gck*-3′-UTR clone containing the wild-type miR-206 seed sequence was halved compared with that transfected with *Gck*-3′-UTR clone with the selectively scrambled miR-206 seed sequence. This confirmed the sequence specificity for miR-206-mediated regulation of *Gck*. In addition, our findings of unaltered insulin content in pancreata of miR-206KO mice demonstrate that increased GSIS in miR-206KO mice is independent of insulin synthesis and regulated by GK activity, which controls intracellular ATP production via glucose 6-phosphate availability for glycolysis and K^+^/Ca^2+^ increase. ATP inhibits the K^+^ ATP channel, leading to a depolarization event causing increased intracellular Ca^2+^ influx, which then directly activates the synaptotagmin 5 and synaptotagmin-like 4 proteins on the insulin granule, triggering the fusion of the insulin granule with the plasma membrane (27).

Defining the exact mechanism of miR-mediated regulation is daunting given the complex target repertoire and target interaction. For example, although GK activity is important for initiating the glycolytic flux, the most crucial enzyme involved is phosphofructokinase/fructobisphosphatase (PFK-1). *Pfk-1* mRNA is not a predicted target of miR-206. However, the most powerful allosteric activator of PFK-1 is fructose-2,6-bisphosphate (Fru-2,6-P2). The levels of Fru-2,6-P2 are regulated by the enzyme fructose-2,6-biphosphatase 3, the mRNA of which is a predicted target of miR-206 (16).

A limitation of our study is the lack of a cell system overexpressing miR-206 to prove the effect of miR-206 overexpression on GK. miR-206 expression in MIN-6 or isolated islets decreases cell viability significantly (unpublished observation). Consistent with our own observations, Liu et al. (13) and others have reported miR-206 as a proapoptotic activator of cell death. Lower levels of baseline (*time 0*) glucose and increased liver glycogen in HFD-fed miR-206KOs are indicative of a role of hepatic miR-206 in regulating systemic glucose levels. In control mice, HFD compared with regular chow leads to a 1.5-fold upregulation in hepatic miR206 expression. The cumulative impact of this upregulation in a metabolically active tissue such as the liver could be significant. As expected, miR-206KOs challenged with HFD showed increased GK activity and higher levels of hepatic glycogen. However, the expected increase in the transcript levels of hepatic *Gck* upon HFD was not observable. This leads us to speculate that miR-206 might have other targets that may mediate its effects on glucose metabolism in a tissue-specific manner. Such tissue-specific effects of hepatic vs. islet miR-206 on glucose metabolism are not discernable in a germline knockout model such as the one used in this study. Therefore, future studies aimed at investigating tissue-specific knockouts of miR-206 and the effects on glucose homeostasis are warranted.

Taken together, we have described the novel posttranscriptional regulation of islet *Gck*. Our data revealed that miR-206 expression is tissue specifically altered in response to HFD, and loss of miR-206 increases GK activity in islets and liver, leading to improved glucose tolerance and GSIS. There is an increasing effort to develop GK activators to treat diabetes. However, in conditions like obesity and diabetes, aberrant expression of posttranscriptional regulators such as miR-206 can repress the levels of GK, making GK activators less effective.

## Figures and Tables

**Table 1:** List of miR-206 targetgenes (shown in red in Fig. 3) that are upregulated in diabetic patient islets compared with nondiabetic control islets

Entry ID	Symbol	Name
Q92922	SMARCC1	SWI/SNF complex subunit SMARCC1
Q16875	PFKFB3	6-Phosphofructo-2-kinase/fructose-2,6-bisphosphatase 3
P53539	FOSB	ProteinfosB
P17535	JUND	Transcription factor jun-D
Q9UGR2	ZC3H7B	Zinc finger CCCH domain-containing protein 7B
Q92841	DDX17	Probable ATP-dependent RNA helicase DDX17
O14617	AP3D1	AP-3 complex subunit-δ1
P24385	CCND1	G1/S-specific cyclin-D1
O95544	NADK	NAD kinase
P56545	CTBP2	C-terminal-binding protein 2
P064545	PTMA	Prothymosin-α
Q14135	VGLL4	Transcription cofactor vestigial-like protein 4
P04085	PDGFA	Platelet-derived growth factor subunit -
Q969W9	PMEPA1	Protein TMEPAI
Q12802	AKAP13	A-kinase anchor protein 13

**Table 2:** List of miR-206 target genes (shown in green in Fig. 3) that are downregulated in diabetic patient islets compared with nondiabetic control islets

Entry ID	Symbol	Name
P17844	DDX5	Probable ATP-dependent RNA helicase DDX5
P61978	HNRNPK	Heterogeneous nuclear ribonucleoprotein K
P63104	YWHAZ	14-3-3 proteinζ/δ
Q14444	CAPRIN1	Caprin-1
P10809	HSPD1	60-kDa heat shock protein, mitochondrial
P43034	PAFAH1B1	Platelet-activating factor acetylhydrolase IB subunit-α
P61224	RAP1B	Ras-related protein Rap-1b
P62995	TRA2B	Transformer-2 protein homolog-β
Q9Y6X1	SERP1	Stress-associated endoplasmic reticulum protein 1
P47813	EIF1AX	Eukaryotic translation initiation factor 1A, X-chromosomal
P21281	ATP6V1B2	V-type proton ATPase subunit B, brain isoform
O00264	PGRMC1	Membrane-associated progesterone receptor component 1
P48444	ARCN1	Coatomer subunit-δ
O43143	DHX15	PremRNA-splicing factor ATP-dependent RNA helicase DHX15
P06730	EIF4E	Eukaryotic translation initiation factor 4E
O75787	ATP6AP2	Renin receptor
P04166	CYB5B	Cytochrome b5 type B
Q13242	SRSF9	Serine/arginine-rich splicing factor 9
P51790	CLCN3	H^+^/Cl^−^) exchange transporter 3
Q12792	TWF1	Twinfilin-1
Q9UGP8	SEC63	Translocation protein SEC63 homolog
Q96CQ1	SLC25A36	Solute carrier family 25 member 36
P38606	ATP6V1A	V-type proton ATPase catalytic subunit A
O60519	CREBL2	cAMP-responsive element-binding protein-like 2
Q14139	UBE4A	Ubiquitin conjugation factor E4 A
O60749	SNX2	Sorting nexin-2
Q9Y2H6	FNDC3A	Fibronectin type-III domain-containing protein 3A
Q9C040	TRIM2	Tripartite motif-containing protein 2
P60880	SNAP25	Synaptosomal-associated protein 25
P04035	HMGCR	3-hydroxy-3-methylglutaryl-coenzyme A reductase
O15155	BET1	BET1 homolog
Q53EL6	PDCD4	Programmed cell death protein 4
P07947	YES1	Tyrosine-protein kinase Yes
O00139	KIF2A	Kinesin-like protein KIF2A
Q9UKA4	AKAP11	A-kinase anchor protein 11
Q9UQR1	ZNF148	Zinc finger protein 148
Q14206	RCAN2	Calcipressin-2
O43617	TRAPPC3	Trafficking protein particle complex subunit 3
Q53HI1	UNC50	Protein unc-50 homolog
Q9Y5K6	CD2AP	CD2-associated protein
P30793	GCH1	GTP cyclohydrolase 1
O75061	DNAJC6	Putative tyrosine-protein phosphatase auxilin
P61604	HSPE1	10-kDa heat shock protein, mitochondrial
Q8N3U4	STAG2	Cohesin subunit SA-2
P84103	SRSF3	Serine/arginine-rich splicing factor 3
Q3UN86	G3BP2	Ras GTPase-activating protein-binding protein 2
P30626	SRI	Sorcin
Q99442	SEC62	Translocation protein SEC62
Q15788	NCOA1	Nuclear receptor coactivator 1
P62253	UBE2G1	Ubiquitin-conjugating enzyme E2 G1
Q92973	TNPO1	Transportin-1
Q99967	CITED2	Cbp/p300-interacting transactivator 2
O14981	BTAF1	TATA-binding protein-associated factor
Q86UL8	MAGI2	Membrane-associated guanylate kinase, WW and PDZ domain-containing protein 2
P20936	RASA1	Ras GTPase-activating protein 1
Q9BUL8	PDCD10	Programmed cell death protein 10
P62158	CALM1	Calmodulin
P40189	IL6ST	Interleukin-6 receptor subunit-β
O14786	NRP1	Neuropilin-1
O14977	AZIN1	Antizyme inhibitor 1
Q15057	ACAP2	Arf-GAP with coiled-coil, ANK repeat and PH domain-containing protein 2
Q13492	PICALM	Phosphatidylinositol-binding clathrin assembly protein
O00571	DDX3X	ATP-dependent RNA helicase DDX3X
P31629	HIVEP2	Transcription factor HIVEP2
Q96AE4	FUBP1	Far upstream element-binding protein 1
Q8IYH5	ZZZ3	ZZ-type zinc finger-containing protein 3
Q9UJ04	TSPYL4	Testis-specific Y-encoded-like protein 4
Q5VWQ0	RSBN1	Round spermatid basic protein 1
P43490	NAMPT	Nicotinamide phosphoribosyltransferase
Q8IYB5	SMAP1	Stromal membrane-associated protein 1
Q9P215	POGK	Pogo transposable element with KRAB domain
P53367	ARFIP1	Arfaptin-1
Q5U5Q3	MEX3C	RNA-binding E3 ubiquitin-protein ligase MEX3C
Q8IVH8	MAP4K3	Mitogen-activated protein kinase kinase kinase kinase 3
Q9H0F5	RNF38	E3 ubiquitin-protein ligase RNF38
Q8WVD3	RNF138	E3 ubiquitin-protein ligase RNF138
Q86UB9	TMEM135	Transmembrane protein 135
Q5MIZ7	SMEK2	Serine/threonine-protein phosphatase 4 regulatory subunit 3B
